# Tissue Culture and Refreshment Techniques for Improvement of Transformation in Local Tetraploid and Diploid Potato with Late Blight Resistance as an Example

**DOI:** 10.3390/plants9060695

**Published:** 2020-05-29

**Authors:** Eu Sheng Wang, Nam Phuong Kieu, Marit Lenman, Erik Andreasson

**Affiliations:** Department of Plant Protection Biology, Swedish University of Agricultural Sciences, SE-230 53 Alnarp, Sweden; Eu.Sheng.Wang@slu.se (E.S.W.); Nam.Kieu.Phuong@slu.se (N.P.K.)

**Keywords:** resistance genes, *Agrobacterium* transformation, recalcitrant, *Phytophthora infestans*, refreshment protocol

## Abstract

Potato (*Solanum tuberosum*) is among the best producers of edible biomass in terms of yield per hectare and a variety of different regional cultivars are used as a staple commodity in many countries. However, this crop is attacked by several diseases, with the worst being the late blight disease caused by *Phytophthora infestans*. Stacking of resistance (R) genes from wild *Solanum* relatives are interesting prospects for the sustainable control of late blight. Therefore, we optimized methods for the efficient generation and screening of R-gene-containing transformants in tetraploid and diploid hybrid potato genotypes. Using these methods, a high transformation efficiency was achieved for the transformation of tetraploid and diploid potato lines with a triple resistance (3R) gene construct. Transformation efficiencies were improved by optimizing several factors affecting regeneration, including the quality of the starting plant material, and the composition of the plant growth regulators used during selective regeneration. A refreshment protocol was designed to alleviate in vitro related stress in stock plants, which significantly improved the growth vigor and resulted in a 4- to 10-fold increase in transformation efficiency. Furthermore, long-term exposure to exogenous Indole-3-butyric acid that is usually used for the initiation of roots in vitro, was found to cause aberrant morphological phenotypes in potato.

## 1. Introduction

Potato is a good staple source of carbohydrate in many countries, even though worldwide and regional differences in cultivar preference exist [1]. One of the main diseases affecting potato today is the late blight disease caused by the oomycete pathogen *Phytophthora infestans*. Late blight was responsible for the destruction of the potato crop in Ireland between 1845 and 1849, which caused the death of more than one million people, through a period of massive starvation known as the ‘Irish Famine’ [2]. Under favorable conditions, without counteractive measures, the entire plant can be destroyed in only a matter of days, with the blight spreading to the rest of the field in just weeks [3].

Resistance (R) genes against *P. infestans* from wild *Solanum* species provide a means for developing late-blight-resistant potato cultivars. R-genes with relatively broad resistance were identified in several wild potato relatives [4], but the stacking of several R-genes is probably still required to attain sustainable resistance levels in the field. However, the highly heterogeneous potato genome structure, tendency for self-incompatibility, and severe inbreeding depression remain as major stumbling blocks for conventional breeding [5]. Interestingly, advancements in hybrid potato breeding using homozygous diploid lines were made by commercial breeding companies, such as Solynta [6]. Hybrid diploid lines with combinations of R-genes are now available, but it is still uncertain how competitive this material will be in the market.

Currently, biotechnology-based stacking of R-genes provides a good solution for sustained late blight control and has the benefit of producing much quicker results, compared to conventional breeding methods [7,8,9]. An efficient and versatile pipeline for the transformation, regeneration, selection, and screening of R-gene transformants is required in order to evaluate their potential usefulness in potato. Such a pipeline would also allow genes for many other traits to be integrated directly into different elite potato lines, and to be field-tested in less than a year.

The T-DNA transfer mechanism of *Agrobacterium tumefaciens* was widely used for the transformation of potato [7,8,10,11]. The efficiency of transformation is variable between different potato genotypes and accessions. The effectiveness of *Agrobacterium* transformation is highly dependent on several factors, including explant age or developmental stage, type of plant organs transformed, regeneration viability, and susceptibility/response to Agrobacteria infection. The quality of the starting plant material is regarded as an important variable [12]. Plant lines that were maintained for an extended duration in vitro, tended to suffer a decline in vitality as they acclimatized to unfavorable in vitro conditions. The high humidity present within the culture vessels, a constant source of exogenous sugars and the extended exposure to plant growth regulators tended to induce epigenetic changes that were reflected by a general decline in growth vigor [13]. Studies on grapevine plants indicated that altered epigenetic DNA methylation patterns and histone modifications, induced by unfavorable in vitro conditions or heat stress, gradually reverted back to normal when the stress conditions were discontinued [14,15]. Interestingly, an improvement in shoot regeneration from transformed leaf explants was also reported in one potato genotype, when ‘fresh explants’ prepared from in vitro micro-tubers were used [16].

In this paper, we report optimized methods for the improvement of potato genotypes using *Agrobacterium*-mediated transformation for the stacking of R-genes. We successfully used these methods for the fast transformation and subsequent screening of a triple resistance (3R) gene construct in tetraploid potato cv. King Edward and an inbred diploid potato line (B101). Techniques for the ‘refreshment’ of in vitro-maintained potato lines were also discussed, together with their merits for improving transformation efficiency in potatoes.

## 2. Results and Discussion

### 2.1. Introduction of a Triple Resistance Gene Stack in Désirée

**Figure 1:**
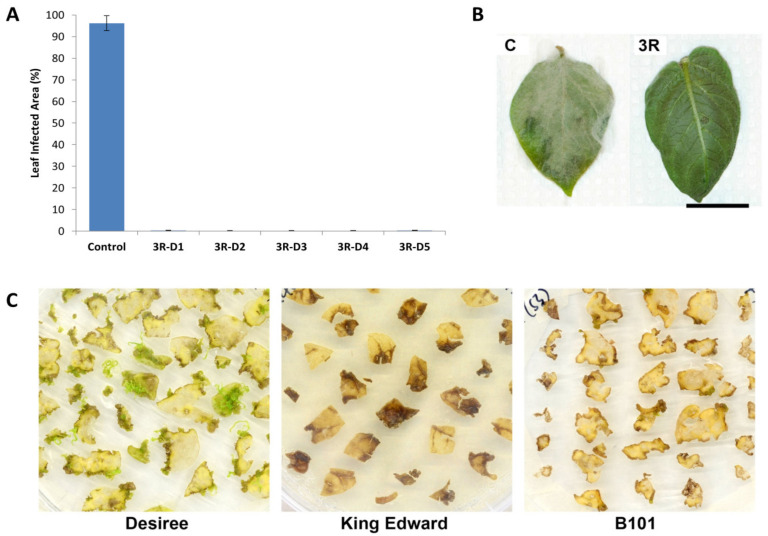
*Solanum tuberosum* cv. Désirée transformed with the triple resistance (3R) gene stack RB, Rpi-blb2 and Rpi-vnt1.1 against *P. infestans* and shoot regeneration. (**A**) Mean leaf affected area for detached-leaf assay for wild-type Désirée (Control) and 3R Désirée (3R-D1, 3R-D2, 3R-D3, 3R-D4, and 3R-D5) against P. infestans at 7 dpi. Results are expressed as mean % leaf area ± standard deviation. Ten leaves were observed for each line and the detached leaf assay was repeated three times. All 3R Désirée lines tested showed a significant difference against the control at *p* < 0.05 through a one-way ANOVA. (**B**) Pictures from the infected leaf from the wild-type Désirée (C = control) and 3R Désirée (3R) in detached-leaf assays. Bar = 20 mm. (**C**) Selective regeneration of resistant shoots from transformed leaf explants of Désirée, King Edward, and B101, using the standard medium and protocols.

The triple resistance (3R) gene construct [9] was transformed into *S. tuberosum* cv. Désirée via *Agrobacterium* transformation. Using the original transformation protocol [10], transgenic shoots of Désirée could be recovered, characterized, and phenotypically tested in the laboratory, within a 4- to 5-month period. The outlined protocol allowed a regeneration efficiency of approximately 45% (total number of resistant shoots recovered over the number of explants transformed) with up to 90% of the regenerated shoots (59 out of the 65 recovered lines) carrying the T-DNA sequence of interest. All resistance genes were covered by the genomic PCR analysis (Figure S1A). We also analyzed an *Agrobacterium* gene in order to verify no contamination of *Agrobacterium* itself (Figure S1A). All recovered 3R-transformed Désirée lines showed enhanced resistance against infection by *P. infestans*. In detached-leaf assays, leaves from 3R-transformed Désirée lines showed practically no infection symptoms at 7 dpi, as compared to their wild-type counterpart, which were completely covered by mycelial growth (Figure 1A,B). This left enough time for the production of seed tubers under greenhouse conditions, followed by a 2- to 3-month cold-treatment period for dormancy breaking before field trials, all within a one-year timeframe.

A reliable pipeline for the transformation of *S. tuberosum* cv. Désirée, followed by its subsequent characterization, phenotypic screening, and field trials within a one-year timeframe was successfully established in our laboratory. This short timeframe was interesting, as it would allow for the development of resistant potato cultivars that had the potential to keep up with the changing pathogen populations. This was also interesting, for example, in a rotation plan of different resistance sources of transgenics to maintain stable resistance against diseases where stacking was not possible. The adaptation of these methods for the improvement of locally popular potato cultivars and pre-breeding material such as inbred diploid potato lines was also of utmost interest. One example is cv. King Edward, which is the most commonly cultivated cultivar in Sweden, and constitutes 20% of the table potato production [1].

In our study, leaf explants from the in vitro maintained cv. King Edward lines were found to be highly susceptible to damage during transformation. Likewise, the near homozygote diploid line, B101, had poor in vitro vigor and the leaf explants were very susceptible to damage during transformation. The damaged tissue of both cv. King Edward and B101 leaf explants rapidly underwent necrosis during the regeneration process, which severely reduced the viability of the surrounding tissue (Figure 1C). Using the original protocol [10], regeneration of cv. King Edward and B101 was much lower compared to cv. Désirée, with only a few shoots emerging from the non-transformed leaf explants after 8–12 weeks (data not shown). Consequently, efforts were made to increase the recovery of transformed shoots in this study by optimizing the use of plant growth regulators during in vitro cultivation and regeneration.

### 2.2. Long-Term Effects of Plant Growth Regulators on In Vitro Plant Material

**Figure 2:**
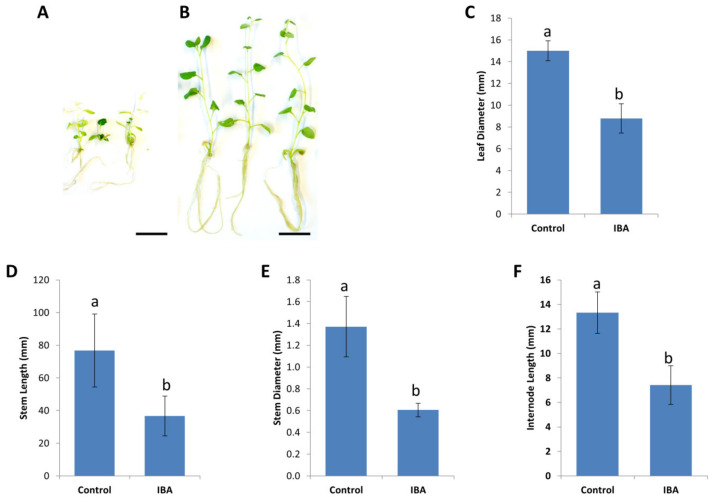
Long-term effects of the auxin Indole-3-butyric acid (IBA) on the morphological and physiological characteristics of *Solanum tuberosum* cv. King Edward in-vitro. (**A**) In-vitro propagated *S. tuberosum* cv. King Edward on media containing 0.2 mg/L IBA displaying aberrant shoot morphologies, after 4 months of in-vitro culture. (**B**) In-vitro propagated *S. tuberosum* cv. King Edward stock lines on agar media containing no IBA, displaying typical shoot morphology. Bar = 20 mm. Effects of IBA on the (**C**) leaf diameter, (**D**) stem length, (**E**) stem diameter, and (**F**) internode length, after 4 months of continuous culture on media containing 0.2 mg/L IBA. Results are expressed as mean ± standard deviation. Thirty explants were observed for each treatment and the experiments were repeated twice. Bars denoted by different lowercase letters a and b indicate a significant difference at *p* < 0.05, through one-way ANOVA for each part of the figure separately.

Usually between 0.1–0.2 mg/L indole-3-butyric acid (IBA) is included in the media used for the propagation of stock plants to induce root development. However, we found the addition of IBA unnecessary, as potato stem cuttings readily formed roots without IBA, albeit about one week later, compared to cuttings grown on media containing 0.2 mg/L IBA (Figure 2). Although King Edward shoots initially produced more root biomass on 0.2 mg/L IBA, these shoots displayed aberrant root morphology, reduced growth vigor, and shortened stems, when subsequently transferred to media without IBA (Figure 2A). Stock plants of cv. King Edward also began to exhibit reduced apical dominance, formed thinner stems and developed smaller leaves, when maintained on media containing 0.2 mg/L IBA for 4 months or longer. These aberrant phenotypes were also observed to a lesser degree at lower IBA concentrations of 0.1 and 0.05 mg/L, although the addition of IBA at these concentrations did not significantly improve root development (data not shown). When King Edward shoots kept on media containing 0.2 mg/L IBA were transferred to media without IBA, the aberrant phenotypes worsened further (Figure 2A). This phenotype was not observed in King Edward shoots that were continuously maintained on media without IBA (Figure 2B). This data suggests that King Edward shoots might alter endogenous auxin homeostasis in response to exogenous IBA causing a “carry-over effect” following transfer to media without IBA, as suggested by [17]. This is not ideal for the maintenance of stock plants for *Agrobacterium* transformation where the leaf material is used. To mitigate any carry-over effects that might hamper regeneration, IBA was not included in the maintenance media of stock plants (Table 1).

### 2.3. Optimization of Plant Growth Regulators for Shoot Induction from King Edward and B101

**Figure 3:**
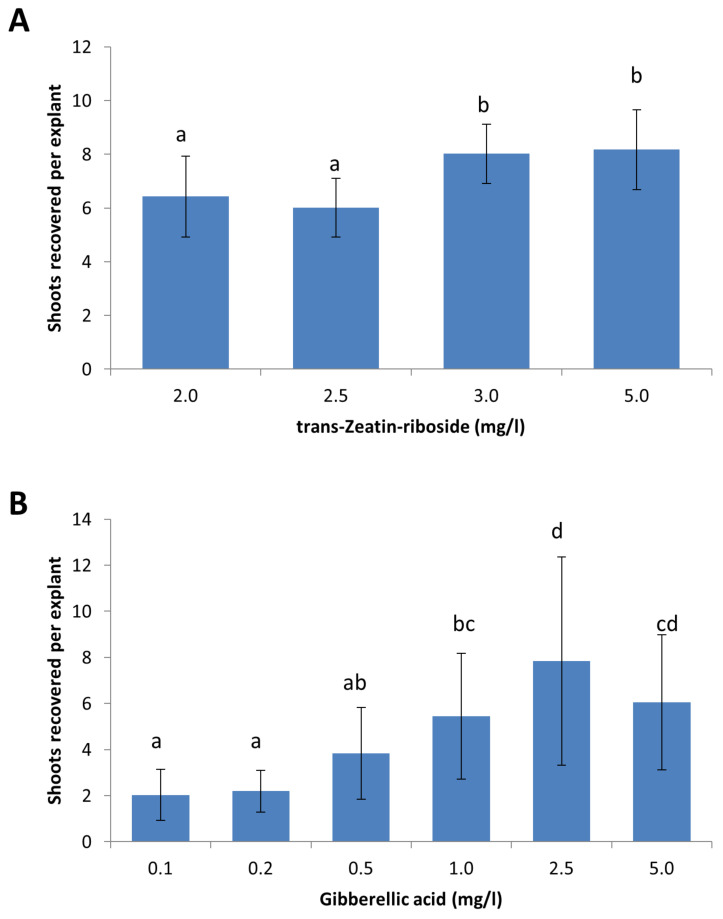
Optimization of plant-growth regulator concentrations for the shoot induction media used for selective regeneration. (**A**) Trans-Zeatin-riboside concentrations were optimized for the induction of shoots from leaf explants of *S. tuberosum* cv. King Edward. (**B**) Gibberellic acid concentrations were optimized to promote shoot development from dormant buds in B101. Results are expressed as mean ± standard deviation. A total of sixty explants were observed for each treatment and the experiments were repeated twice. Treatments denoted by different lowercase letters a–d indicate a significant difference at *p* < 0.05, by Tukey’s Honest Significant Difference test for each part of the figure separately.

Earlier transformation experiments using the selection and regeneration protocol for *S. tuberosum* cv. Désirée did not yield satisfactory results for *S. tuberosum* cv. King Edward and B101 (Figure 1C). Although callus induction was observed at the cut edges of King Edward leaf explants following regeneration on media containing 2.0 mg/L BAP and 0.2 mg/L NAA, the callus failed to form buds and differentiate into shoots. The cytokinin trans-Zeatin-riboside is often used in plant tissue cultures to stimulate plant cell division and to induce shoot formation [18]. The effects of increasing the concentration of trans-Zeatin-riboside in the regeneration media was evaluated at concentrations of 2.0 mg/L, 2.5 mg/L, 3.0 mg/L, and 5.0 mg/L (Figure 3A). The results indicated a general increase in shoot induction frequencies at higher concentrations of trans-Zeatin-riboside, with concentrations of 3.0 mg/L and 5.0 mg/L giving the best results with 8.0 and 8.2 recovered shoots per explant, respectively. The difference was significant (*p* < 0.05) between these higher concentrations, as compared to 2.0 mg/L and 2.5 mg/L, where an average of 6.4 and 6.0 shoots were recovered per explant, respectively. The concentration of 4.0 mg/L trans-Zeatin-riboside was used for the selective regeneration of King Edward leaf explants.

In contrast to *S. tuberosum* cv. King Edward, the cut leaf edges of transformed B101 leaf explants readily formed callus and buds. However, the buds that formed were dormant and did not develop further to form shoots (data not shown). Gibberellic acid (GA3) is a plant growth regulator that stimulates plant growth and development, and is known to trigger transitions from the meristem to shoot growth [19,20,21]. Therefore, several concentrations of GA3 (0.1 mg/L, 0.2 mg/L, 0.5 mg/L, 1.0 mg/L, 2.5 mg/L, and 5.0 mg/L) were tested in the shoot induction media for B101. The highest shoot induction frequency was observed with a GA3 concentration of 2.5 mg/L, which corresponded to the average recovery of 7.8 shoots per explant. This was significantly different from the results obtained using GA3 concentrations 0.1 mg/L, 0.2 mg/L, 0.5 mg/L, and 1.0 mg/L (*p* < 0.05), but was not significantly different from the results of 5.0 mg/L GA3 (Figure 3B). The concentration of 2.5 mg/L GA3 was chosen as the optimum concentration for the selective regeneration of B101 as the average number of shoots recovered per explant using 2.5 mg/L GA3, was higher than 5.0 mg/L (7.8 versus 6.0) (Figure 3B). Furthermore, shoots that were initiated on shoot induction media containing 5.0 mg/L GA3 also appeared thinner and displayed a higher frequency of aberrant phenotypes, as compared to shoots that were recovered from shoot induction media containing 2.5 mg/L GA3 (data not shown).

A summary of the optimized media used for the selective regeneration of putative transformants of *S. tuberosum* cv. Désirée, King Edward and B101 is shown in Table 1. In brief, a higher level of trans-Zeatin-riboside of at least 3.0 mg/L was important for the induction of buds from callus material in the King Edward leaf explants. The developed buds then readily differentiated into shoots, after 4 to 6 weeks on the shoot induction media. For B101, the callus material was able to develop buds that remained dormant and required elevated levels of GA3 to promote shoot development. The optimal concentration of GA3 was determined to be 2.5 mg/L for the regeneration of shoots from B101.

### 2.4. Improving Regeneration with Refreshed Stock Lines

In our laboratory, potato lines were propagated in vitro to provide starting material for *Agrobacterium*-mediated transformation. In general, plant material that is maintained in vitro for extended periods tend to show aberrant morphological and physiological changes. These might include the formation of smaller leaves with lower chlorophyll content, lower chloroplast numbers, formation of thinner stems, and the loss of apical dominance [13,14]. In order to improve in vitro plant vigor, and thereby provide better starting material for transformation experiments, a ‘refreshment’ protocol was developed and integrated as part of the maintenance routine.

**Figure 4:**
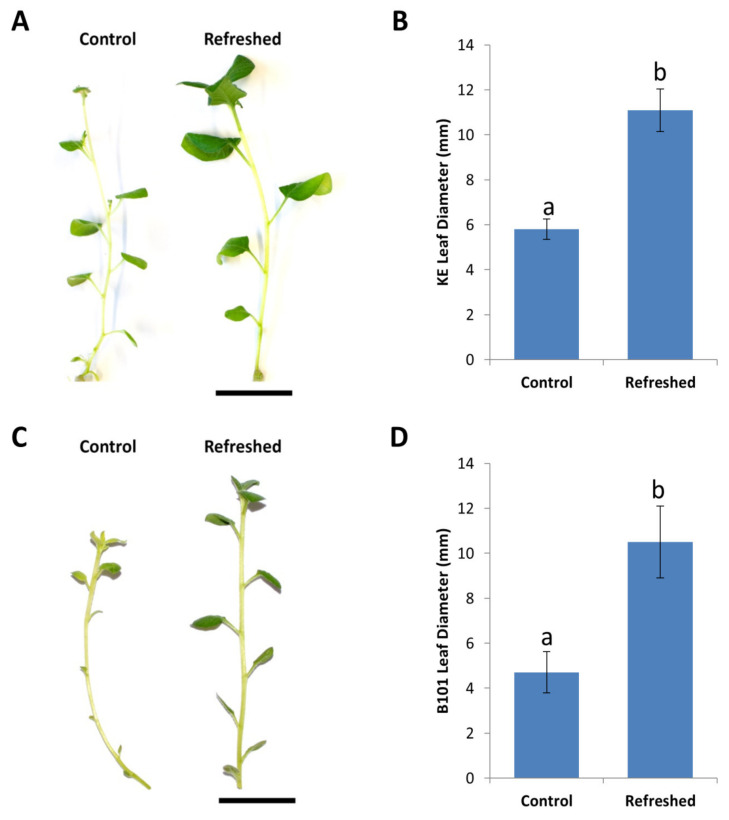
Improvement of plant vigor in *Solanum tuberosum* cv. King Edward and B101, following refreshment ex vitro on soil and in vitro via mini-tubers, respectively. (**A**) King Edward shoots showing improved shoot vigor following refreshment treatments ex vitro. (**B**) Increase in leaf diameter following refreshment treatment in King Edward (KE). (**C**) Refreshed B101 shoots displaying improved growth vigor following in vitro refreshment via mini-tubers. (**D**) B101 lines producing larger leaves following refreshment as compared to controls. Results are expressed as mean ± standard deviation. Twenty shoots were examined from each of the refreshed and control treatments and the experiment was repeated three times. Bars denoted by different lowercase letters a and b indicate a significant difference at *p* < 0.05 through one-way ANOVA for each part of the figure separately.

Potato lines were refreshed by using either one of two methods. The first method involved the acclimatization of in vitro plants ex vitro for 1–2 months, followed by the reintroduction of surface sterilized lateral buds back in in vitro. The second method involves the induction of new shoots from mini-tubers that are produced from in vitro maintained stock lines. Refreshed potato lines displayed higher growth vigor, significantly larger leaves and a decline in aberrant morphological and physiological phenotypes, as compared to the controls (Figure 4A,C). Leaves were 1.9× larger in the refreshed King Edward (Figure 4A,B) and 2.2× larger in the refreshed B101 (Figure 4C,D).

**Figure 5:**
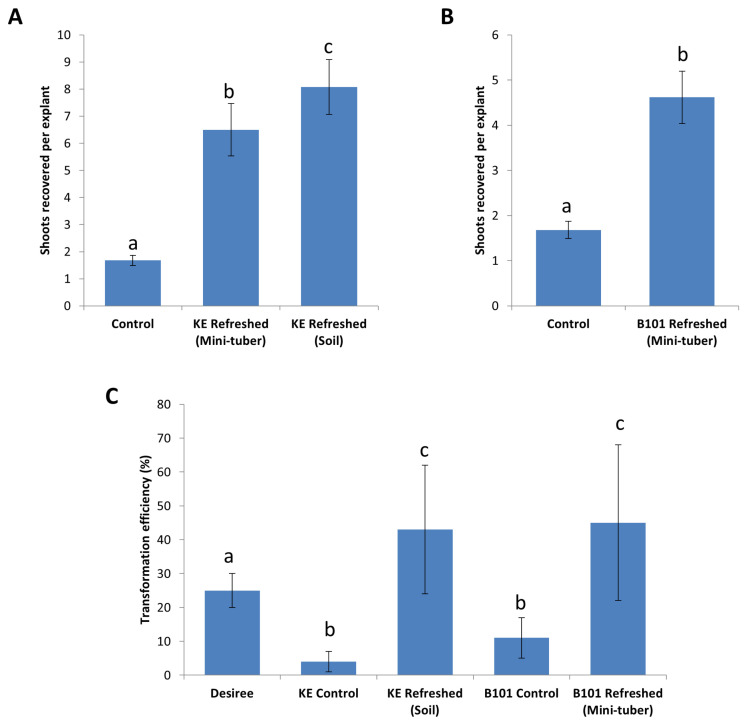
Improvement of shoot induction frequencies by refreshing in vitro stock lines on soil (ex vitro) and via mini-tubers (in-vitro). (**A**) Stock lines of *Solanum tuberosum* cv. King Edward (KE) refreshed on soil and via mini-tubers showing a higher shoot induction frequencies leading to a higher number of shoots recovered per explant as compared to the control lines. (**B**) Stock lines of diploid B101 refreshed via mini-tubers showing a higher shoot induction frequencies on SIM compared to controls. (**C**) Refreshed lines of both *S. tuberosum* cv. King Edward (KE) and B101 with higher growth vigor contributed to a higher recovery of transformed shoots during *Agrobacterium* transformation with the 3R construct. Results are expressed as mean ± standard deviation. A total of sixty explants were observed for each treatment and the experiments were repeated twice. Treatments denoted by different lowercase letters a–c indicate a significant difference at *p* < 0.05 by the Tukey’s Honest Significant Difference test for each part of the figure separately.

The improvement in growth vigor contributed to an increase in the number of shoots recovered from leaf explants of King Edward and B101, during regeneration on shoot induction media (Figure 5A). The ex vitro method for refreshment (on soil) provided the best results for *Solanum tuberosum* cv. King Edward where shoot recovery was increased 4.8-fold from 1.7 shoots per leaf explant to 8.1 shoots. Refreshment via mini-tubers improved the recovery of shoots by 3.9-fold, corresponding to the average recovery of 6.5 shoots per explant. For B101, in vitro maintained stock lines were susceptible to abiotic stresses and could not easily be refreshed on soil and, therefore, in vitro refreshment via mini-tubers was chosen. Refreshment of B101 lines via mini-tubers improved shoot regeneration 2.7-fold, corresponding to an average recovery of 4.6 shoots per leaf explant, as compared to 1.7 in the controls (Figure 5B). Thus, we concluded that both refreshment methods improved shoot induction of King Edward and B101 on SIM, with the ex vitro method being slightly more effective for King Edward. In order to test if this general increase in shoot induction efficiency was reflected in an improved recovery of transgenic shoots, we transformed the 3R construct previously used for Désirée into leaf explants of both King Edward and B101.

### 2.5. Stacking of Resistance Genes in King Edward and B101

We transformed leaf explants of both King Edward and B101 with the 3R gene construct using the same *Agrobacterium* transformation protocol previously used for Désirée, and subjected the explants to a 48 h co-cultivation period, post-transformation. Selective regeneration of transformed King Edward and B101 leaf explants were performed using the respective media that were optimized here (summarized in Table 1). Leaf explants from refreshed lines of both King Edward and B101 showed a significant improvement in regeneration performance. An approximate 10.8-fold increase in shoot induction was observed in the refreshed King Edward leaf explants transformed with the 3R construct, corresponding to a transformation efficiency of 43% (140 putative transformants out of the 322 transformed leaf explants, which were positive for the 3R insert during PCR-screening). This was a significant increase over the control lines which only had a transformation efficiency of 4% (*p* = 2.5 × 10^−8^) (Figure 5C). An approximate 4.1-fold increase was also observed for the transformation of refreshed B101 leaf explants transformed with the 3R construct, resulting in a transformation efficiency of 45% (98 putative transformants screened positive for the 3R insert out of the 220 transformed leaf explants). This difference was also significantly higher than the control lines, which showed a transformation efficiency of 11% (*p* = 0.025) (Figure 5C). The results supported the notion that the quality of the starting material is an important factor to be considered for the improvement of transformation efficiencies. Thus, the periodic refreshment of in vitro lines was highly beneficial for increasing plant consistency, vigor, and transformation success. This was in agreement with the findings by [16] in *S. tuberosum* cv. Atlantic, where ‘fresh explants’ were prepared using a similar in vitro method.

**Figure 6:**
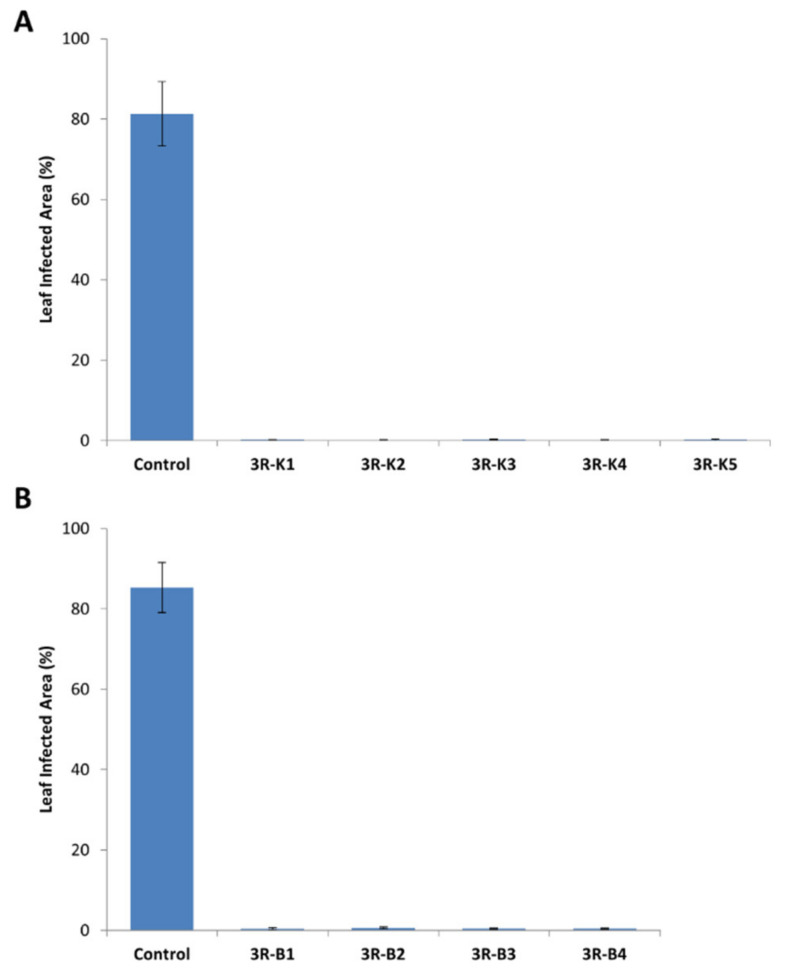
Evaluation of transgenic King Edward and B101 lines transformed with the triple resistance (3R: RB, Rpi-blb2, and Rpi-vnt1.1) gene stack against *P. infestans* in detached-leaf assays. (**A**) Detached-leaf assay results for wild-type King Edward (Control) and the 3R-transformed King Edward lines (3R-K1 to K5) at 7 dpi. (**B**) Detached-leaf assay results for wild-type B101 (Control) and 3R-transformed B101 lines (3R-B1 to B5) at 7 dpi. Results are expressed as mean ± standard deviation. Ten leaves were observed for each line and the detached-leaf assay was repeated three times. All 3R lines tested showed a significant difference against the control at *p* < 0.05, through one-way ANOVA.

Similar to Désirée, all selected 3R-transformed King Edward and B101 lines contained all three resistance genes without *Agrobacterium* contamination (Figure S1A). Expression of all three resistance genes was also observed in the recovered King Edward lines (Figure S1B). Resistance levels of these lines against *P. infestans* were tested using the same detached-leaf assay, as described earlier for Désirée (Figure 6). Similar to 3R Désirée, results from the detach-leaf assays for the 3R-transformed King Edward and B101 also displayed resistance against *P. infestans* infection, as compared to wild-type controls at 7 dpi (Figure 6A,B). These results indicate that a similar signal transduction mechanism exist in Désirée, King Edward, and B101, which allowed the R-genes to recognize the presence of the corresponding avirulence genes of *P. infestans* and elicit an effective defense response.

The timeline for the entire process of transformation, regeneration, molecular characterization, and screening, under optimized conditions for King Edward and B101 took a total of 4 to 5 months, matching that of the model cultivar Désirée. This was interesting especially in the inbred diploid line, B101, since potato is known to suffer from inbreeding depression [22]. Using our pipeline, we demonstrated that B101 could also be readily transformed and express similar levels of resistance as the tetraploid lines in infection assays. Therefore, we propose that our transformation methods are probably versatile and can be used for the transformation of a variety of potato material, including locally popular tetraploid cultivars, such as King Edward and inbred diploid lines such as B101.

## 3. Materials and Methods

### 3.1. Materials

The *P. infestans* strain 88069 was propagated, as described in [23]. The diploid inbred potato line B101 was obtained from seeds of Solynta (Wageningen, The Netherlands). The line was developed in a diploid hybrid potato-breeding program, as described in [6]. The inbred line did not contain any known resistance to late blight. In vitro stocks of diploid line B101 and tetraploid *S. tuberosum* cvs. Désirée and King Edward were maintained by subculturing the apical portion of 3–4 week old stems on standard Murashige and Skoog (MS) media (20 g/L sucrose, 4 g/L phyto agar, pH 5.8), in a tissue culture box (Phytatray, Sigma, Missouri, USA) [24]. All media and chemicals used for the in vitro work were sourced from Duchefa Biochemie (Haarlem, The Netherlands), unless stated otherwise. The 3R transformation vector (pCIP99) containing three resistance genes (*Rpi-blb2*, *RB* (*Rpi-blb1)* and *Rpi-vnt1.1*) was provided by Marc Ghislain, as described in [9].

### 3.2. Potato Transformation Protocol

The protocol for the *Agrobacterium* transformation of *S. tuberosum* cv. Désirée was essentially performed, according to the protocol by Visser [10], while protocols for cv. King Edward and the diploid potato line B101 were modified. A total of 250 leaf explants were transformed in each transformation cycle, with 25 explants maintained per 90 mm Petri dish. The transformation cycles were repeated until a total of 100 antibiotic resistant shoots were recovered for molecular screening. For transformation, an overnight 10-ml liquid culture of *Agrobacterium* C58 carrying the plasmid of interest was centrifuged, at 5000 g for 10 min, to pellet the bacteria. The supernatant was discarded and the pellet was rinsed gently and re-suspended in 10 mL of dH2O. Prior to transformation, 100 µL of acetosyringone (76 mM) was added to the *Agrobacterium* suspension. For transformation, 1 mL of the *Agrobacterium* suspension (OD 1.0) was pipetted onto the dissected leaf explants that were placed on the callus inducing media (CIM) (Table 1). Transformed leaf explants were incubated at 25 °C, 16/8 h light/dark photoperiod at 25 μmol.m^2^·s^−1^ (50% intensity) for 48 h, before they were transferred to shoot inducing media (SIM) for selective regeneration (Table 1). The CIM and SIM media used for selective regeneration of cv. King Edward and B101 were modified, as described in Table 1. Leaf explants were subcultured on fresh SIM every 2 weeks to maintain selection pressure, and all plates were incubated at 25 °C, 16/8 h light/dark photoperiod, at 50 μmol.m^2^·s^−1^. Shoots that emerged after 4–5 weeks were dissected and rooted on MS1, containing no plant growth regulators, but with a continued selection (100 mg/L kanamycin and 400 mg/L cefotaxime, Table 1). Only shoots that initiated roots in the selective media were further screened at the molecular level.

### 3.3. Molecular Screening

Genomic DNA was extracted from 100–200 mg of young leaves from in vitro regenerated shoots [25], and used as template for PCR analysis. The PCR reaction mixture contained 1× Buffer, 2 µL (200 ng) genomic DNA, 0.2 mM dNTPs, 0.5 μM of each primer, and 0.2 U Taq DNA polymerase (Thermo Fisher Scientific, Waltham, USA), to a final volume of 25 µL. All primers, amplicon lengths, as well as corresponding templates analyzed are shown in Table S1. The PCR amplification program for genomic DNA was as follows—1 cycle of 5 min at 95 °C followed by 35 cycles of denaturation (20 s at 94 °C), annealing (20 s at 62 °C), and extension (30 s at 72 °C), and a final extension at 72 °C for 5 min. The samples were analyzed on 1% agarose gels. RNA extraction and cDNA synthesis was performed, as described in [26]. In brief, RNA was extracted with Qiagen RNeasy mini kits (Qiagen, Limburg, the Netherlands), according to the manufacturer’s instructions, and 500 ng total RNA were transcribed to cDNA, using the SuperScript III Reverse Transcriptase, including degradation with RNase H, according to the manufacturer’s protocol (InvitroGen, Carlsbad, CA, USA). PCR amplifications were essentially done as described above, except that 30 rounds of amplification were used instead (see Table S1 for annealing temperature). Products were run on 2.5% agarose gels.

### 3.4. Maintenance and Refreshment of In Vitro Material

Plant lines were maintained in vitro by subculturing the top 15–20 mm portion of the apical shoot onto fresh full-strength MS media every 3–4 weeks (Table 1). All in vitro plant material were incubated in a culture chamber at 25 °C, 50% relative humidity, 16/8 h light/dark photoperiod at 50 μmol.m^2^·s^−1^. Refreshment of plant lines was performed using either an ex vitro or in vitro method. For the ex vitro method, young in vitro shoots that had just begun to initiate roots were transferred to potting soil and grown in a controlled environment for 1–2 months [27]. Lateral buds were dissected from the adult plants, surface-sterilized, and reintroduced back in vitro according to [27]. After 7 days, any contaminated material was removed, and after 14 days, the non-contaminated shoots that remained were treated as sterile and used for the establishment of new refreshed in vitro stock lines. For the in vitro refreshment method, 20 mm apical shoots were cultured on full-strength MS media (30g/l sucrose, pH 8) in the culture chamber, under a 16/8 h light/dark photoperiod at 50 μmol.m^2^·s^−1^ for 6 to 8 weeks, without subculture, to allow the development of mini-tubers in vitro. The mini-tubers were collected, dissected half lengthwise, and placed cut-side-down on MS media (20 g/L sucrose, pH 8) containing 2.0 mg/L BAP for the induction of shoot development. Shoots that emerged 2–3 weeks later were dissected and transferred to full-strength MS media (Table 1) and amplified to produce a refreshed stock line. Any morphological differences between the refreshed and control (non-refreshed) lines were recorded and the data were analyzed through one-way analysis of variance (ANOVA), with the significance level calculated at *p* ≤ 0.05.

### 3.5. Detached-Leaf Assay

To generate plant material for the detached-leaf assay, in vitro generated plants were grown in potting soil in a controlled environment [28]. The inoculum of *P. infestans* spores was prepared by harvesting sporangia from 12- to 14-day old plates of *P. infestans* in clean tap water [29] and was adjusted to 50,000 sporangia/ml. Zoospores were released by cold treatment before inoculation [30]. For infection assays, 25 µL of the spore solution was pipetted onto the abaxial side of the leaflet. The infected leaves were kept in a humid environment (RH ~100%) under controlled conditions [31] for 7 days. Results were recorded by measuring the diameter of the infection zone on each individual leaflet, 7 days post-inoculation (dpi). A total of 9 leaflets per line were used for the detached-leaf assay. Data were collected from 3 technical replicates and analyzed using one-way analysis of variance (ANOVA) with significance calculated at *p* ≤ 0.05.

### 3.6. Data Collection and Statistical Analysis

All physical measurements were made using a standard pair of Vernier calipers. For the detached-leaf assays, the infected area was calculated as a percentage of the total leaf area to accommodate for any discrepancies in leaf size and to provide a more accurate comparison of resistances between different lines. Statistical analyses were performed using one-way ANOVA, and Tukey’s Honest Significant Difference test, when a comparison between more than 2 groups was required. Transformation efficiencies were calculated based on the total number of transgenic shoots positive for the 3R construct in molecular screening assays, over the total number of explants transformed.

## Figures and Tables

**Table 1:** Media Composition for the maintenance, transformation, selection, and regeneration of Désirée, King Edward, and the B101 plant material. CIM = Callus induction media; SIM = Shoot induction media. In bold are major optimizations suggested from this investigation.

	Plant Material			
Components	Désirée	King Edward	B101	Use
MS1				Maintenance of in vitro lines, rooting media, stock propagation
MS salts + Vitamins	4.4 g/L	4.4 g/L	4.4 g/L	
Sucrose	10 g/L	10 g/L	10 g/L	
pH	5.8	5.8	5.8	
Phytoagar	8 g/L	8 g/L	8 g/L	
CIM (MS1 including compounds below)				Co-cultivation post transformation, callus induction
BAP	2.0 mg/L	2.0 mg/L	2.0 mg/L	
trans-Zeatin-riboside	-	-	-	
NAA	0.2 mg/L	0.2 mg/L	0.2 mg/L	
GA_3_	-	-	-	
SIM (MS1 including compounds below)				Selection and regeneration post transformation, shoot induction
BAP	-	-	-	
trans-Zeatin-riboside	2.0 mg/L	4.0 mg/L	2.0 mg/L	
NAA	0.01 mg/L	0.01 mg/L	-	
GA_3_	0.1 mg/L	0.1 mg/L	2.5 mg/L	
Kanamycin	100 mg/L	100 mg/L	100 mg/L	
Claforan (cefotaxime)	400 mg/L	400 mg/L	400 mg/L	

## Supplementary Materials

The following are available online at https://www.mdpi.com/2223-7747/9/6/695/s1.
