# Supplementary material for: Tissue Culture and Refreshment Techniques for Improvement of Transformation in Local Tetraploid and Diploid Potato with Late Blight Resistance as an Example
Source: Plants (Basel). 2020 May 29;9(6):695. doi: 10.3390/plants9060695 (PMC7356882; doi:10.3390/plants9060695)
Supplement: Supplementary file 1 [file plants-09-00695-s001.pdf]

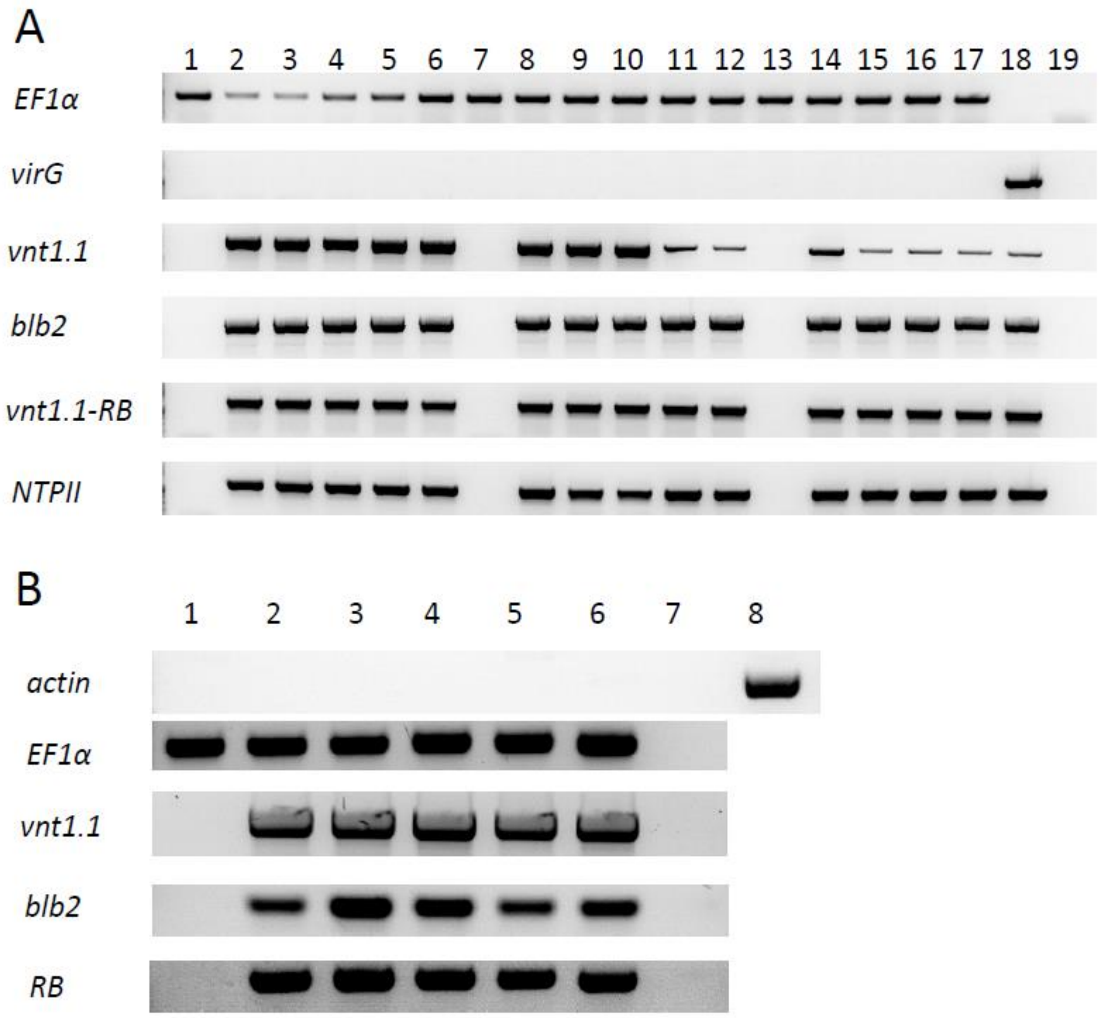

Figure S1. (**A**): PCR results to confirm the presence of the 3 R-gene construct in transgenic lines on genomic DNA. Specific primer pairs (see Table S1) were used to amplify a house keeping gene in potato genome (EF1α), an *Agrobacterium* gene (virG) and genes in in T-DNA construct (vnt1.1, blb2, NTPII and vnt1.1-RB). Genomic DNA templates were 1: Désirée WT, 2–6: Désirée 3R 1 to 5, 7: King Edward WT, 8–12: King Edward 3R 1 to 5, 13: B101 WT, 14-17: B101 3R 1 to 4, 18: *Agrobacterium* cells contains pCIP99-3R, 19: negative control with no genomic DNA added. (**B**) PCR results from cDNA to confirm the expression of all three R-genes in King Edward 3R transgenic lines (vnt1.1, Blb2 and RB). The potato actin_gene_X55750 was used as a negative control, to identify genomic DNA contamination (primer pair spanning an intron-exon border). EF1α as a house keeping gene in potato (positive control). Templates were 1: King Edward WT, 2–6: King Edward 3R 1 to 5, 7: no cDNA added, 8: postive control for actin, King Edward genomic DNA.

Table S1. Primers used in this study.

Template	Primer Name	Primer sequence	Fragment length, bp	Anneali ng temp. (°C)
gDNA/cDNA	St Ef1α F2	GAACTGTCCCTGTTGGTCGT	220	60
	St Ef1α R2	GGGTCATCCTTGGAGTTTGA		
gDNA	VirG+	CGCACGCGCAAGGCAACC		60
	VirG−	GCCGGGGCGAGACCATAGG	606	
gDNA/cDNA	Vnt1.1_R1	GTAAGAGTCAACGGCCCAAG		60
	Vnt1.1_F1_5UT	CCAAACTCACAGCCATGAAC	568	
gDNA	blb2_R1	ATCCTTCTGGCCAAGGATCT	758	60
	blb2_F2	AGTTGCAAGTGCTGTTCACG		
gDNA	Rpi-vnt1.1 RB-F	GCTGCGTTAATTATTTACAT	587	55
	Rpi-vnt1.1 RB-R	GTTGGTTGATTACTTGAACT		
gDNA	LBd_NPTII_F	TGACGAGTTCTTCTGAGCGG		60
	LBd_NPTII_R	CAACTTAATAACACATTGCGGACG	309	
cDNA	qRT_RB-F	CACGAGTGCCCTTTTCTGAC	214	47
	qRT-RB-R	ACAATTGAATTTTTAGACTT		
cDNA	qRT-Rpi-blb2F	TTCAAAACCCCAAATAAGTTTCAAC	81	55
	qRT-Rpiblb2-R	CCATGCTTGCTGTACTTTGCA		
cDNA/gDNA	St PoAc71 F2043i (actin)	TTGTTGGGCTCCTACCAAAG	177	60
	St PoAc71 R2219 (actin)	GAGGGGCCAGACTCATCATA		
